# Elevated plasma abscisic acid is associated with asymptomatic falciparum malaria and with IgG-/caspase-1-dependent immunity in *Plasmodium yoelii*-infected mice

**DOI:** 10.1038/s41598-018-27073-1

**Published:** 2018-06-11

**Authors:** Elizabeth K. K. Glennon, Dewi Megawati, Brandi K. Torrevillas, Isaac Ssewanyana, Liusheng Huang, Fran Aweeka, Bryan Greenhouse, L. Garry Adams, Shirley Luckhart

**Affiliations:** 1 Department of Medical Microbiology and Immunology, University of California Davis, Davis, California United States of America; 2 Infectious Disease Research Collaboration, Kampala, Uganda; 3 London School of Hygiene and Tropical Medicine, London, United Kingdom; 4 Drug Research Unit, Department of Clinical Pharmacology, University of California San Francisco, San Francisco, California United States of America; 5 University of California San Francisco, San Francisco, California United States of America; 6 Department of Veterinary Pathobiology, College of Veterinary Medicine and Biomedical Sciences, Texas A&M University, College Station, Texas United States of America; 7 Present Address: Center for Infectious Disease Research, Seattle, Washington, United States of America; 8 Present Address: Department of Entomology, Plant Pathology and Nematology and Department of Biological Sciences, University of Idaho, Moscow, Idaho United States of America

## Abstract

Abscisic acid (ABA) is an ancient stress hormone and is detectable in a wide variety of organisms where it regulates innate immunity and inflammation. Previously, we showed that oral supplementation with ABA decreased parasitemia in a mouse model of malaria, decreased liver and spleen pathology and reduced parasite transmission to mosquitoes. Here, we report that higher circulating ABA levels were associated with a reduced risk of symptomatic malaria in a cohort of *Plasmodium falciparum*-infected Ugandan children. To understand possible mechanisms of ABA protection in malaria, we returned to our mouse model to show that ABA effects on *Plasmodium yoelii* 17XNL infection were accompanied by minimal effects on complete blood count and blood chemistry analytes, suggesting a benefit to host health. In addition, orally delivered ABA induced patterns of gene expression in mouse liver and spleen that suggested enhancement of host anti-parasite defenses. To test these inferences, we utilized passive immunization and knockout mice to demonstrate that ABA supplementation increases circulating levels of protective, parasite-specific IgG and requires caspase-1 to reduce parasitemia. Collectively, ABA induces host responses that ameliorate infection and disease in an animal model and suggest that further studies of ABA in the context of human malaria are warranted.

## Introduction

Little is currently known about the effects of abscisic acid (ABA) on innate and adaptive immunity to infection and less yet about the mechanisms underlying these effects. Treatment with ABA has been shown to activate granulocytes, monocytes, and microglial cells *in vitro*, increasing phagocytosis, production of reactive oxygen species (ROS) and nitric oxide (NO), enhancing nuclear factor (NF)-κB nuclear translocation and release of monocyte chemotactic protein (MCP-1) and tumor necrosis factor α (TNFα)^1–4^. Interestingly, ABA decreased TNFα expression in white adipose tissue in a model of inflammatory bowel disease and reduced MCP-1 expression and leukocyte infiltration in the lungs of mice with influenza^5,6^. Taken together, these data suggest that the effects of ABA on inflammatory signaling are organ- and context-dependent.

The generalized immune response to *Plasmodium* infection is characterized by a switch from an early pro-inflammatory response to an anti-inflammatory response near peak parasitemia, followed by antibody-mediated parasite clearance. During *P*. *yoelii* infection, IgM antibodies are transiently produced, with protective IgG antibodies (e.g., IgG1, IgG2b) becoming predominant later in infection^7–9^. The innate immune response to *Plasmodium* infection includes clearance of infected red blood cells (iRBCs) by macrophages and dendritic cells in the liver and spleen as well as pro-inflammatory cytokine production following inflammasome activation and signaling through Toll-like receptors and NF-κB^10^. Detection of hemozoin and parasite genomic DNA within the hepatocyte phagolysosome can activate the inflammasome leading to increased production of interleukin (IL)-18 and IL-1β, which are toxic to the parasite^11^. Macrophages secrete IL-1β, interferon γ (IFNγ), and TNFα, which enhance intrahepatic killing of parasites and induce natural killer (NK) cell cytotoxicity^12–14^. TNFα is necessary for IFNγ production by NK cells and is associated with resolution of fever during malaria^15^.

Reciprocal induction of pro-inflammatory cytokine secretion in positive feedback loops can lead to an overactive immune response and immunopathology in malaria^16^. In this context, peroxisome proliferator-activated receptor gamma (PPARγ) is an important regulator of immunity and inflammation. PPARγ agonists have been proposed as malaria therapeutics and can increase CD36-mediated phagocytosis of iRBCs while reducing malaria-induced TNFα secretion^17–19^. ABA can enhance PPARγ levels, and ABA-associated reductions in inflammation were observed to be PPARγ-dependent in inflammatory bowel disease and influenza^5,20^. Similarly, in lipopolysaccharide (LPS)-challenged mice, ABA decreased splenic expression of *il6*, *tnfα*, and *ifnγ* in a PPARγ-dependent manner^21^. We previously demonstrated that ABA supplementation in *P*. *yoelii* 17XNL-infected CD-1 mice reduced parasitemia while increasing *pparγ* expression and decreasing NO synthase (*nos*) expression in both liver and spleen^22^.

Here, we report that Ugandan children with asymptomatic falciparum malaria (no fever) had higher circulating plasma ABA levels than did children with symptomatic disease (fever at presentation). Based on these observations, we returned to our genetically tractable model to investigate possible mechanisms of ABA action during *Plasmodium* infection. We show that orally delivered ABA did not alter complete blood cell count and clinical chemistry analytes in uninfected mice and improved some of these parameters during *P*. *yoelii* 17XNL infection. Further, the beneficial effects of elevated plasma ABA on *P*. *yoelii* 17XNL infection were dependent on caspase-1 and on increased production of IgG, suggesting that elevated plasma ABA may be clinically relevant and protective in falciparum malaria.

## Results

### Reduced risk of symptomatic malaria in *P*. *falciparum* infected children was associated with higher plasma ABA levels

We quantified ABA levels as previously described^22^ in 122 plasma samples from *P*. *falciparum* infected children in Uganda. Children were selected from the Nagongera and Kanungu surveillance cohorts of the East Africa International Center of Excellence for Malaria Research (ICEMR). These cohorts were established in 2011 for health monitoring, including regular visits every 30–90 days during which blood smears and plasma samples were obtained regardless of symptoms^23^. Plasma samples from parasitemic children ages 2–4 and 7–9 years were selected randomly with stratification by age and parasite density.

**Figure 1:**
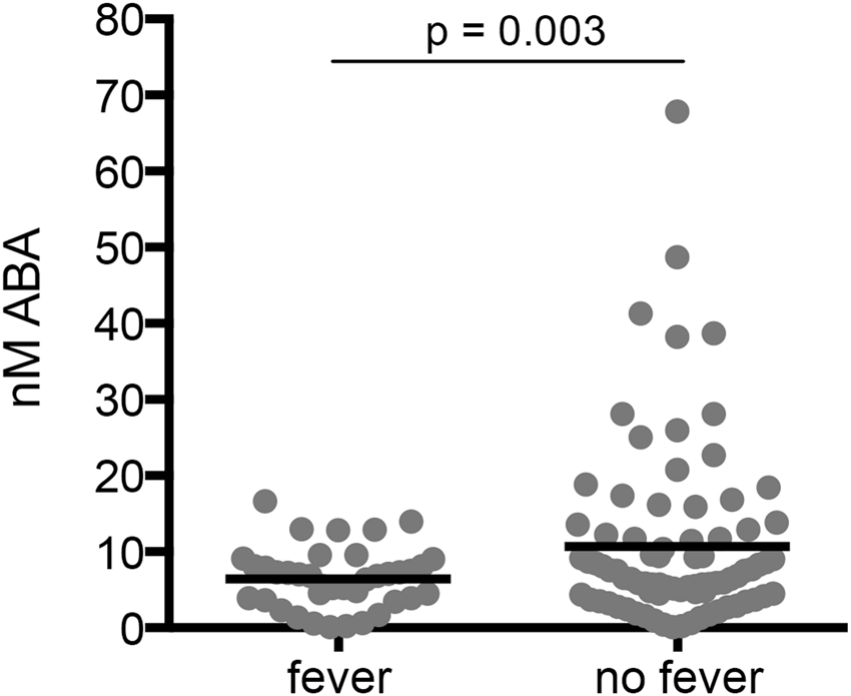
Asymptomatic falciparum malaria in children was associated with higher plasma ABA levels. ABA levels in plasma samples from children, ages 2–9, with detectable *P*. *falciparum* parasitemia. Only children without fever (asymptomatic malaria) had plasma ABA concentrations in excess of 20 nM. Each dot represents one individual (n = 122; 39 with fever, 83 without fever). Data were analyzed by Welch two sample t-test (p = 0.003).

Children with higher plasma ABA levels had asymptomatic infections, not presenting with fever (Fig. 1). These patterns remained evident when samples were stratified by age group (Supplementary Fig. 1), however ABA levels did not correlate with parasitemia (Supplementary Fig. 1). Importantly, plasma levels in children with falciparum malaria were in the same range (<1–100 nM) as detected previously in our mouse model^22^.

### ABA supplementation reduced disease and improved blood urea nitrogen levels in *P*. *yoelii* 17XNL*-*infected C57BL/6 mice

**Figure 2:**
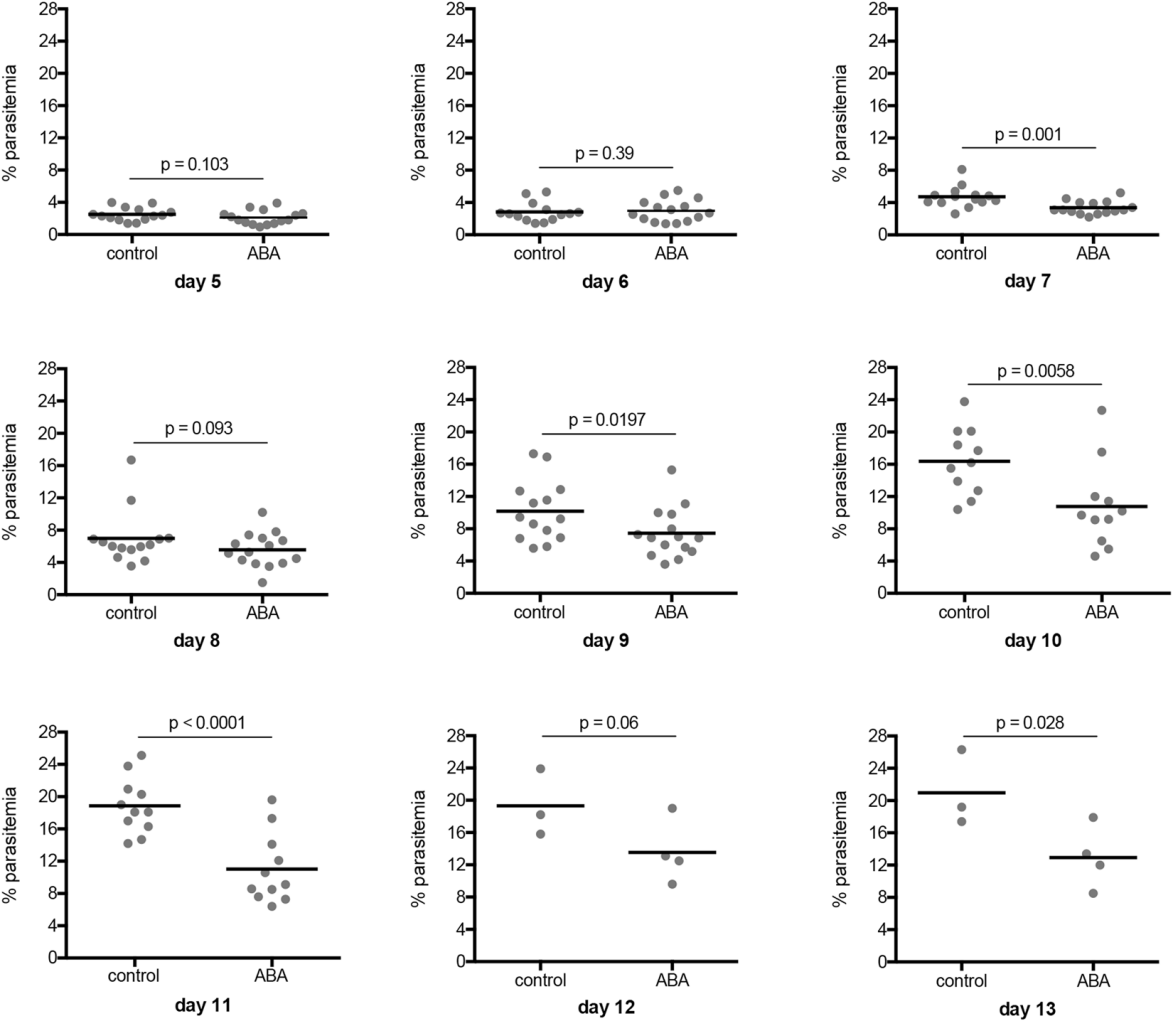
ABA supplementation significantly reduced parasitemia in C57BL/6 mice infected with *P*. *yoelii* 17XNL. Daily parasitemias of mice with and without oral ABA supplementation. Four replicates were conducted with 3–5 mice per treatment in each replicate. Mouse cohorts were sacrificed for analyses on days 9, 11 and 13, reducing animal numbers after these days. Data were analyzed by unpaired t-test.

Our previous studies utilized outbred mice (CD-1 strain) to examine the effects of ABA on *P*. *yoelii* 17XNL parasitemia, pathology and transmission^22^. Here, we established a model for mechanistic studies with C57BL/6 mice. As in our studies with CD-1 mice, 6–8 week old C57BL/6 mice received water containing 2.56 mM ABA for three days prior to and throughout the course of infection with *P*. *yoelii* 17XNL. In four separate cohorts of C57BL/6 mice, we affirmed that ABA supplementation significantly reduced parasitemia (Fig. 2, Supplementary Fig. 2) compared to unsupplemented mice.

**Figure 3:**
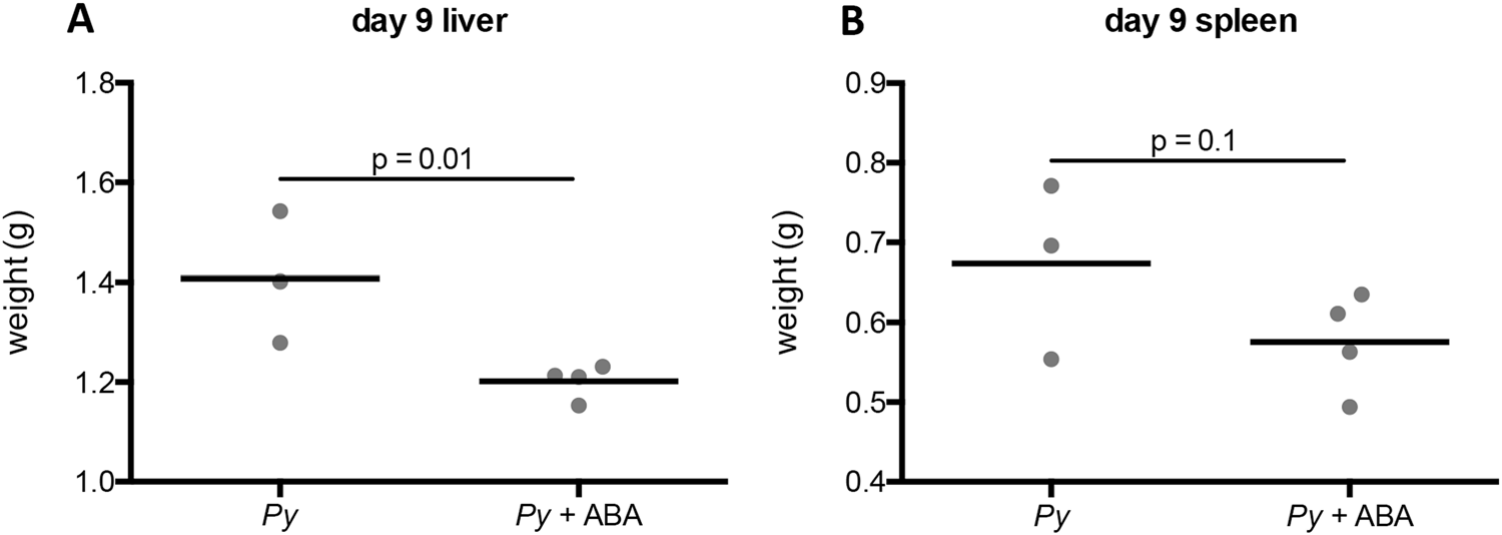
ABA supplemented mice have reduced hepatosplenomegaly. Weights of whole liver (**A**) and whole spleen (**B**) at day 9 PI from *P*. *yoelii* 17XNL-infected mice with and without ABA supplementation. Each dot represents one mouse sacrificed from a single cohort from the data represented in Fig. 2. Data were analyzed by unpaired t-test.

At day 9 post-infection (PI), all infected mice exhibited hepatomegaly and splenomegaly typical of malaria (not shown)^24^. Livers of ABA supplemented mice, however, weighed significantly less than those of unsupplemented mice (Fig. 3A), and there was a trend towards reduced spleen weight with ABA supplementation (Fig. 3B). This was consistent with our previous findings that ABA reduced inflammatory pathology, including microabscesses and leukocyte infiltration in the liver and hyperplasia in the spleen during *P*. *yoelii* 17XNL infection^22^.

**Figure 4:**
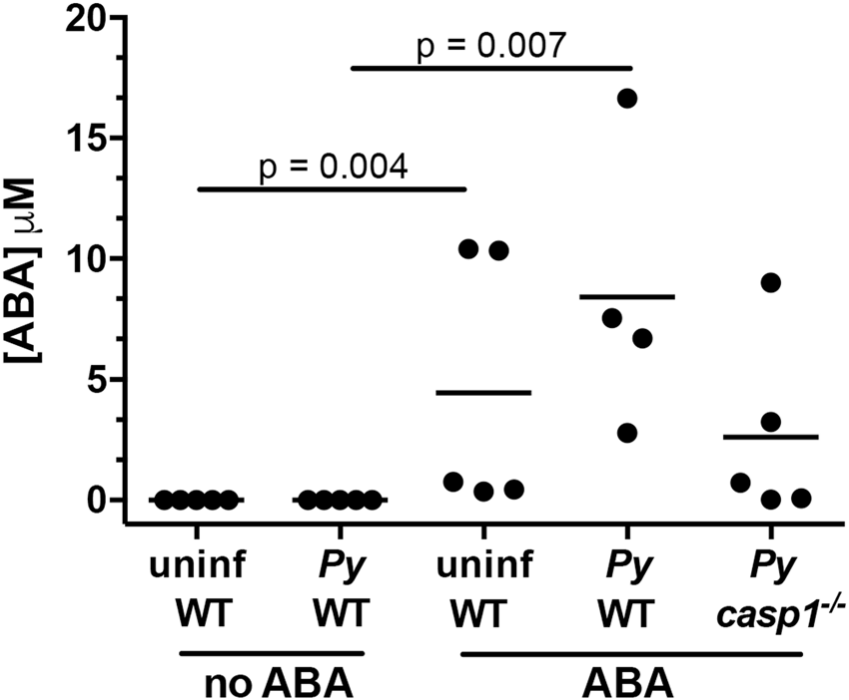
Oral ABA supplementation increased circulating levels of ABA in mouse plasma. ABA concentrations, measured by LC-MS/MS, in plasma of uninfected and *P*. *yoelii* 17XNL-infected wild type C57BL/6 (WT) and *caspase-1* knockout (*casp1*^−/−^) mice with and without ABA supplementation. Plasma samples were collected on day 11 PI. Each dot represents one mouse sacrificed from those represented in Fig. 2. Data were analyzed by unpaired t-test.

Mouse plasma was analyzed for ABA concentration by liquid chromatography-tandem mass spectrometry (LC-MS/MS, University of California San Francisco Drug Research Unit) and for a variety of analytical markers of overall health (University of California Davis Comparative Pathology Lab). Plasma ABA levels were elevated by daily *ad libitum* supplementation in the presence and absence of infection (day 10 PI), with variation likely resulting from patterns and timing of water consumption (Fig. 4). Relative to previous observations^22^, supplemented infected C57BL/6 mice exhibited notably higher levels of plasma ABA than did supplemented CD-1 mice at a similar stage of infection, perhaps due to differences in mouse strain, enhanced sensitivity of LC-MS/MS versus ELISA or both.

**Figure 5:**
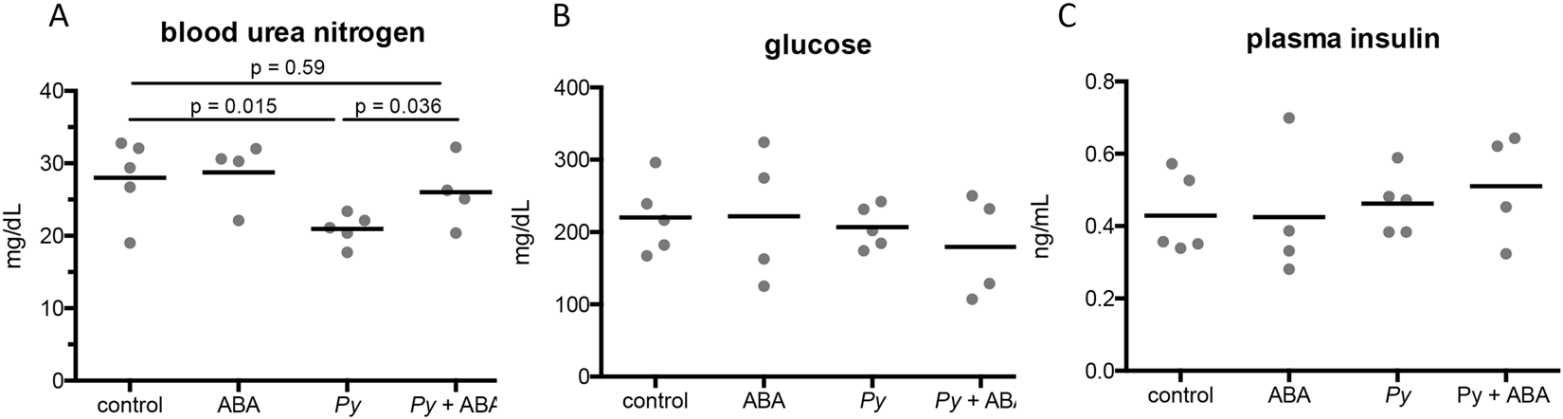
ABA supplementation rescued blood urea nitrogen levels in *P*. *yoelii* 17XNL-infected mice but did not alter glucose or insulin levels in plasma. Blood urea nitrogen (**A**) glucose (**B**), and insulin levels (**C**) in plasma of uninfected mice and *P*. *yoelii* 17XNL*-*infected mice (day 11 PI) with and without ABA supplementation. Each dot represents one mouse sacrificed from those represented in Fig. 2. Data were analyzed by unpaired t-test.

In the presence and absence of infection, ABA supplementation had no effect on levels of plasma albumin, creatine, phosphorus, bilirubin, or total protein (Supplementary Table S1). Infected mice had significantly lower blood urea nitrogen (BUN) levels than uninfected mice (Fig. 5A). ABA supplementation had no effect on BUN levels in uninfected mice but, in the context of infection, ABA-treated mice had plasma BUN levels that were not significantly different from those in uninfected mice (Fig. 5A). Although increased BUN levels have been associated with kidney damage, abnormally low levels can also be indicative of impaired liver function^25–27^, suggesting that the effects of ABA extend beyond resolution of liver histopathology to improved function.

### ABA supplementation had no effect on baseline blood glucose or insulin levels

ABA supplementation has been shown to increase glucose-potentiated insulin release and has been proposed as an adjunctive treatment for type-2 diabetes^28,29^. Because hypoglycemia and hyperinsulinemia are pathological symptoms associated with malaria^30^, we sought to explore the potential for ABA to exacerbate these conditions. Neither baseline glucose nor insulin levels, however, were significantly different from controls after two weeks of oral ABA supplementation in uninfected or *P*. *yoelii* 17XNL*-*infected C57BL/6 mice (day 11 PI, Fig. 5B,C).

### ABA increased the proportion of eosinophils and basophils in blood of *P*. *yoelii* 17XNL*-*infected C57BL/6 mice

**Figure 6:**
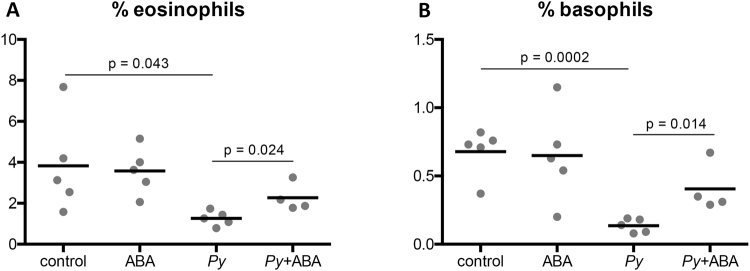
ABA supplementation partially rescued the decrease in eosinophils and basophils associated with *P*. *yoelii* 17XNL infection. Percentage of eosinophils (**A**) and basophils (**B**) in whole blood of uninfected mice and *P*. *yoelii* 17XNL*-*infected mice (day 11 PI) with and without ABA supplementation. Each dot represents one mouse sacrificed from those represented in Fig. 2. Data were analyzed by unpaired t-test.

Complete blood counts (CBC, University of California Davis Comparative Pathology Lab) were performed on whole blood from uninfected and *P*. *yoelii* 17XNL-infected mice (day 11 PI) with and without ABA supplementation. In the absence of infection, ABA had no effect on blood cell composition (percentages of neutrophils, lymphocytes, monocytes, eosinophils, basophils, and platelets), hemoglobin levels, or percent hematocrit (Supplementary Table S2). In the presence of infection, the percentages of eosinophils and basophils were reduced and ABA significantly increased these to levels approaching those in uninfected mice (Fig. 6). Though little is known about the role that eosinophils and basophils play in malaria, eosinophilia has been associated with recovery from infection^31,32^. No other changes in blood cells or hemoglobin values were observed with ABA supplementation (Supplementary Table S2).

### Gene expression patterns associated with ABA supplementation were suggestive of inflammasome activation and antibody isotype switching in *P*. *yoelii* 17XNL*-*infected mice

**Figure 7:**
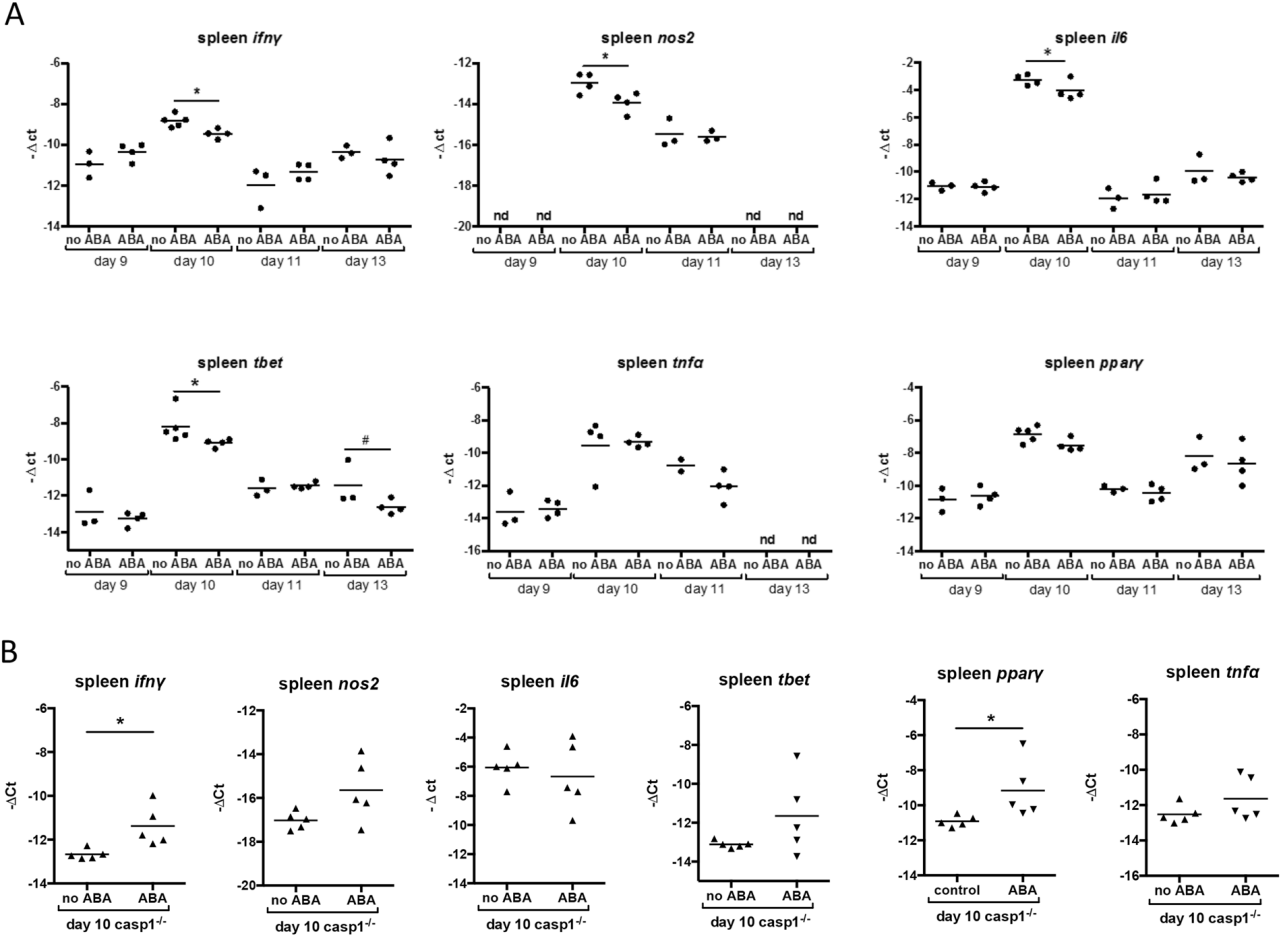
Splenic gene expression patterns following ABA supplementation were suggestive of enhanced IgG production. Relative target gene mRNA levels in spleens of unsupplemented and ABA-supplemented C57BL/6 mice on days 9, 10, 11, and 13 PI (**A**) and in *casp1*^−/−^ mice on day 10 PI with *P*. *yoelii* 17XNL (**B**). Each dot represents one mouse with time points in panel (A) collated from three separate cohorts of mice and in panel (B) from one cohort of mice. Data are shown as −Δct, normalized to *β-actin*, and were analyzed by unpaired t-test. *p < 0.05, ^#^p < 0.1.

We monitored spleen and liver gene expression from days 9–13 PI in C57BL/6 mice, the period corresponding to the greatest reductions in parasitemia following ABA supplementation (Fig. 2, Supplementary Fig. 2). We compared gene expression in these tissues to that in the same tissues in CD-1 mice at days 4–7 PI, reflecting our previous observations that the effects of ABA on parasitemia occurred earlier in this mouse strain^22^. In spleen of C57BL/6 mice, expression levels of *ifnγ*, *nos2*, *il6*, and *tbet* were decreased in ABA-supplemented mice at day 10 PI. A trend towards decreased *tbet* was also evident at day 13 PI (Fig. 7A). No change in *tnfα* expression was evident at any timepoint. These patterns were consistent with those in spleen of CD-1 mice at days 4–7 PI (Supplementary Fig. S3A)^22^.

**Figure 8:**
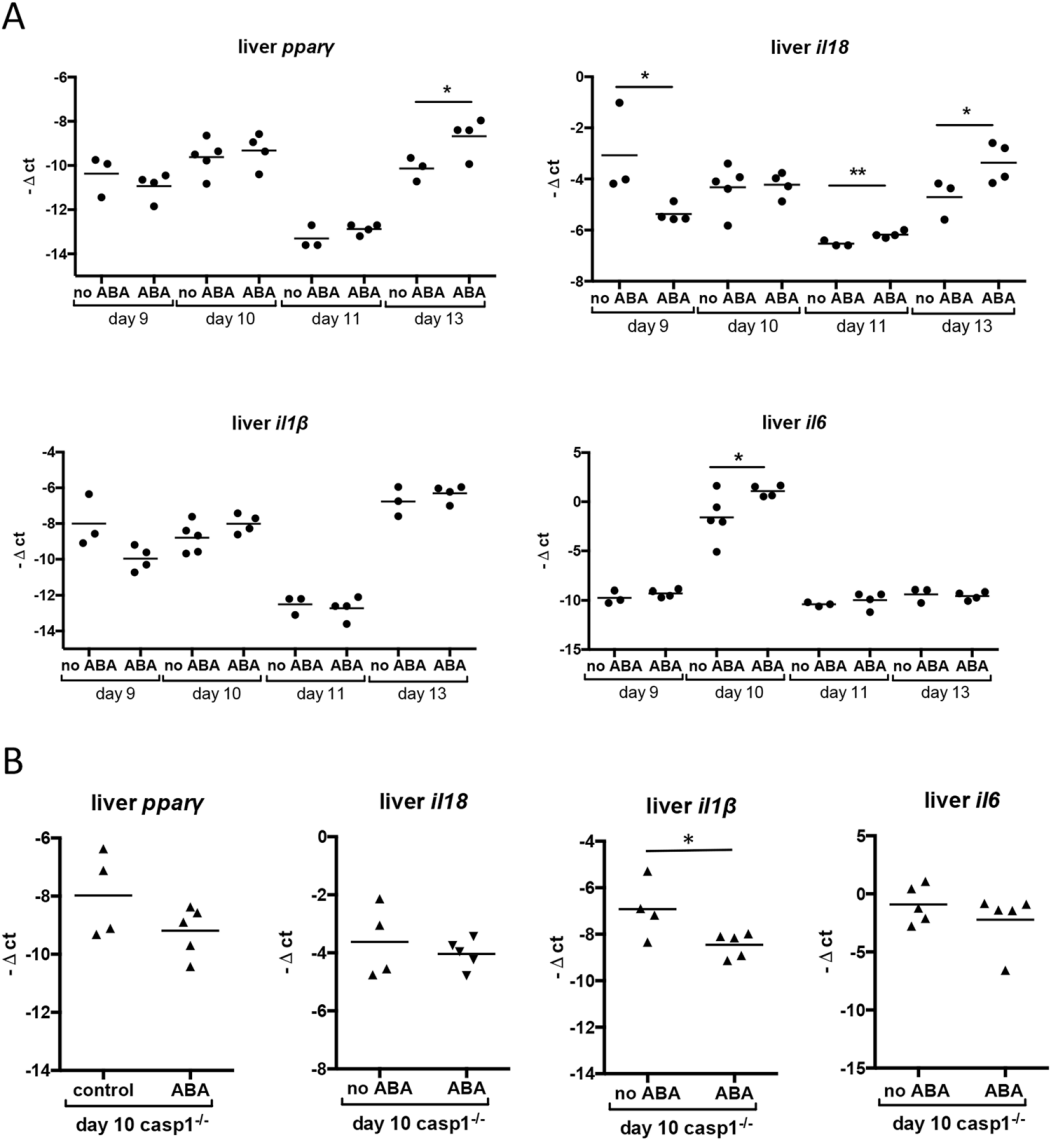
Hepatic gene expression patterns following ABA supplementation were suggestive of inflammasome activation and consistent with reduced hepatomegaly. Relative target gene mRNA levels in livers of unsupplemented and ABA-supplemented C57BL/6 mice on days 9, 10, 11, and 13 PI (**A**) and in *casp1*^−/−^ mice on day 10 PI with *P*. *yoelii* 17XNL (**B**). Each dot represents one mouse with time points collated from the cohorts of mice as in Fig. 7. Data are shown as −Δct, normalized to *β-actin*, and were analyzed by unpaired t-test. *p < 0.05, **p < 0.01.

In livers of C57BL/6 mice, a significant increase in *pparγ* expression was evident by day 13 PI (Fig. 8A). *Il*1*8* expression was significantly lower in ABA-treated mice on day 9 but increased compared to controls on days 11 and 13 PI. *Il1β* mRNA levels did not change significantly at any timepoint while *il6* expression increased on day 10 PI (Fig. 8A). Increased expression of *il18* has been associated with inflammasome activation^33,34^ and contributes to parasite killing in the liver^35^. IL-6 can function in concert with IL-1β to induce the synthesis of specific acute phase proteins^36^. However, IL-6 can also signal through an alternate pathway to promote regenerative and anti-inflammatory signaling in the liver^37–39^, effects that would be consistent with reduced parasitemia in ABA-treated mice as well as reduced hepatomegaly at day 9 PI (Fig. 3A) and recovery of BUN in ABA-treated, infected mice to control levels at day 11 PI (Fig. 5A). ABA-induced gene expression in the liver was similar to patterns in CD-1 mice (Supplementary Fig. S3B)^22^, although increases in *il6* and *il1β* in the liver were more pronounced in ABA-treated, infected CD-1 mice (Supplementary Fig. S3B). Collectively, the consistency of effects of ABA on parasitemia, hepatosplenomegaly, and gene expression patterns in spleen and liver of ABA-treated, infected C57BL/6 and CD-1 mice suggested that ABA effects were robust across divergent mouse models and provided good confidence for testing hypotheses in the more tractable inbred strain.

### ABA supplementation increased levels of parasite-specific, protective circulating IgG antibodies in P. yoelii 17XNL-infected C57BL/6 mice

Based on gene expression patterns in the spleen (Fig. 7A, Supplementary Fig. S3A), we hypothesized that ABA supplementation could alter antibody synthesis and perhaps isotype patterns in infected mice. Specifically, reduced *tbet* expression in B cells can increase total IgG synthesis while increased *tbet* expression correlates with reduced IgG1 production^40,41^. Additionally, low levels of IFNγ can enhance IgG1 synthesis^42^, suggesting that ABA-induced repression of *tbet* and *ifnγ* in the spleen at day 10 PI (Fig. 7A) could increase total IgG and IgG1 in *P*. *yoelii* 17XNL-infected mice.

**Figure 9:**
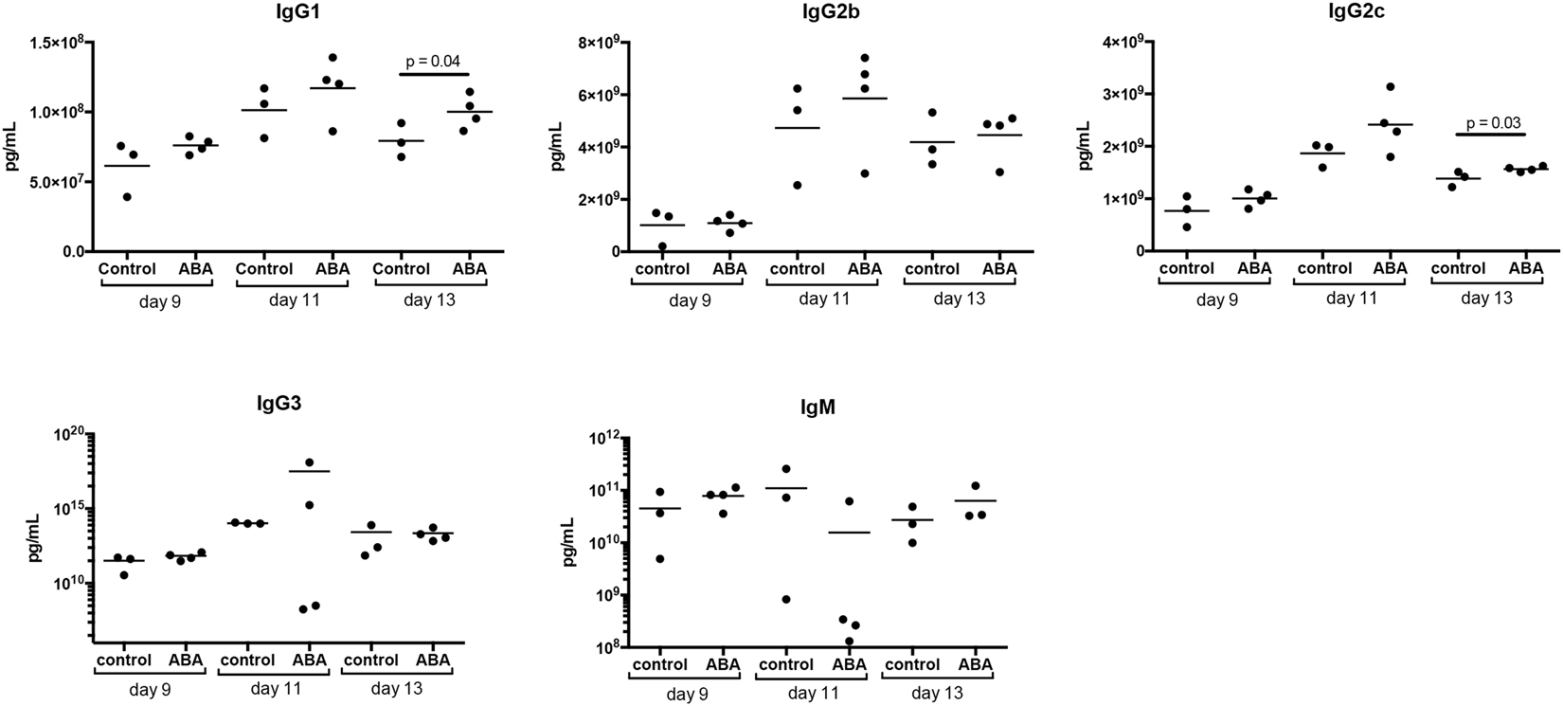
ABA supplementation increased levels of IgG1 and IgG2c in plasma during *P*. *yoelii* infection. Data are shown as concentrations of IgM and IgG antibody isotypes in plasma from mice with and without ABA supplementation on days 9, 11 and 13 PI with *P*. *yoelii* 17XNL. Each dot represents one mouse with time points collated from the cohorts of mice as in Fig. 7. Data were analyzed by unpaired t-test.

**Figure 10:**
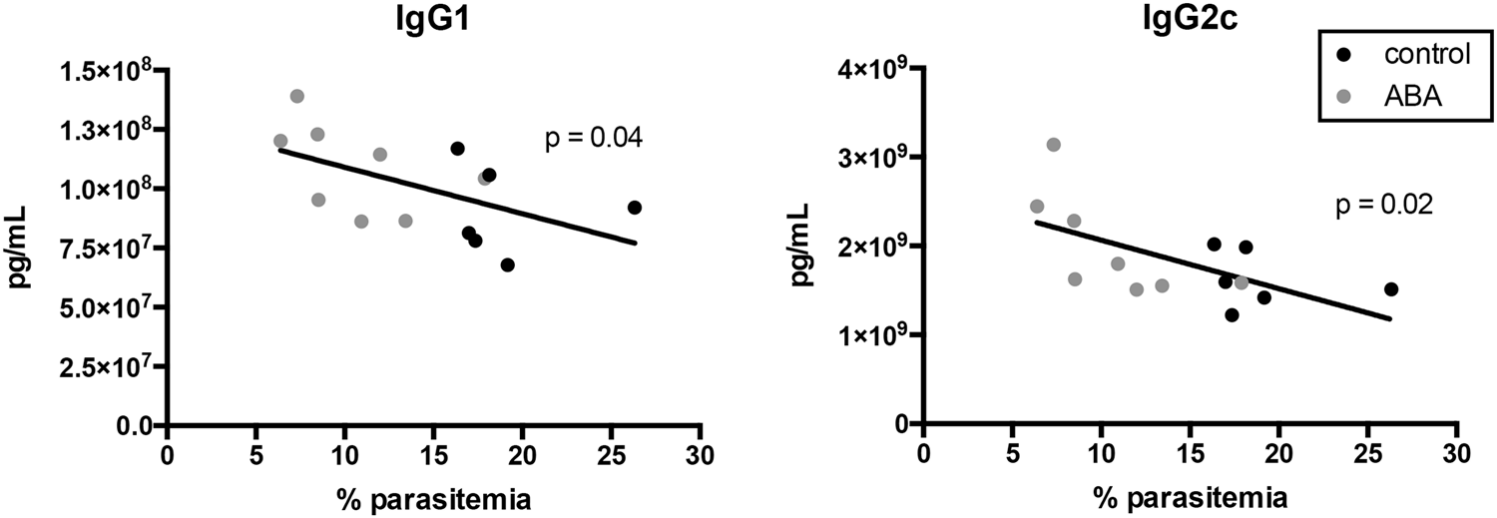
Levels of IgG1 and IgG2c were negatively correlated with parasitemia. IgG1 and IgG2c levels were plotted against parasitemia (days 11 and 13 PI from Fig. 9) from *P*. *yoelii* 17XNL*-*infected mice with and without ABA supplementation. Each dot represents one mouse. Data were analyzed by linear regression.

To determine whether these patterns were evident in our model, we measured antibody levels in plasma from control and ABA-supplemented *P*. *yoelii* 17XNL-infected C57BL/6 mice on days 9, 11, and 13 PI. Levels of IgG1 and IgG2c in ABA-supplemented mice were significantly greater than control levels by day 13 PI, with no changes noted in other isotypes (Fig. 9). Parasitemia and antibody titers were significantly negatively correlated, with lower parasitemias and higher levels of IgG1 and IgG2c in ABA-supplemented mice (Fig. 10).

**Figure 11:**
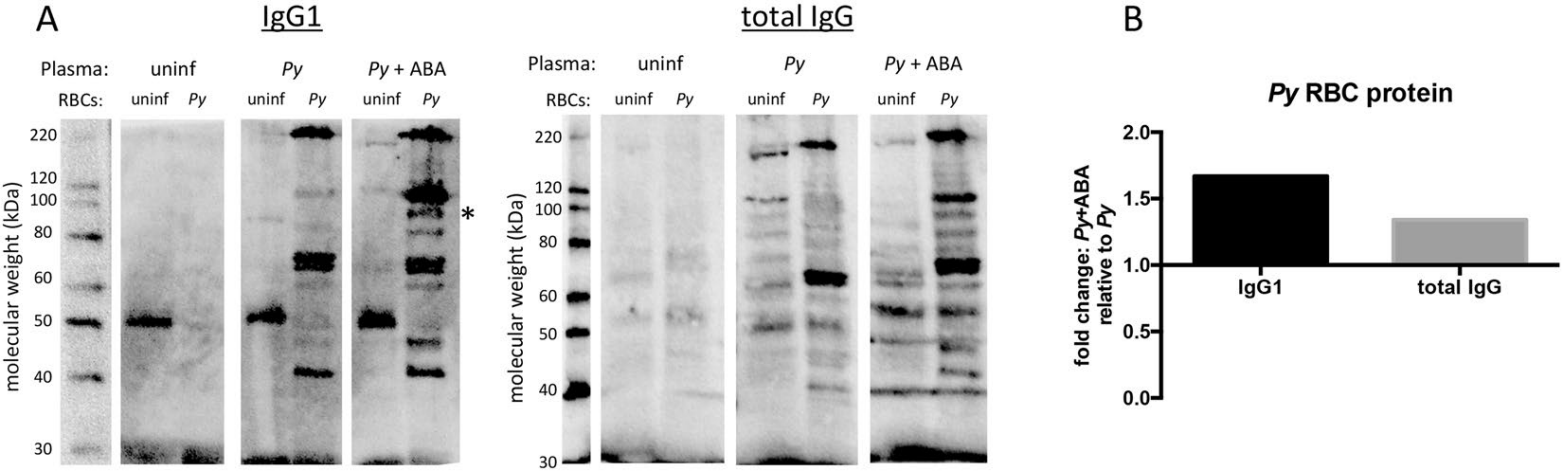
ABA supplementation increased levels of parasite-specific IgG1 and total IgG in mouse plasma. (**A**) Western blots of protein from uninfected and *P*. *yoelii* 17XNL*-*infected RBCs probed with plasma from uninfected mice, *P*. *yoelii-*infected mice or ABA-supplemented *P*. *yoelii-*infected mice followed by secondary antibodies to IgG1 and total IgG. The 50 kDa band corresponds to the IgG heavy chain. Asterisk indicates unique protein (~90 kDa) bound by IgG1 from ABA-treated, infected mice. Samples for IgG and IgG1 detection were each run on a single gel. Membranes were cut for incubation with each type of plasma. Total IgG- and IgG1-probed blots were exposed together and images were cropped to remove white space and redundant protein ladders. (**B**) Densitometry analysis of IgG1- and total IgG-bound proteins from iRBCs. Data are shown as fold change in plasma from ABA-supplemented *P*. *yoelii*-infected mice relative to plasma from unsupplemented, infected mice.

To determine whether increased circulating total IgG and the protective isotype IgG1, which was elevated to a greater degree than protective IgG2c^43^ (Fig. 9), were parasite-specific, we examined the cross-reactivity of plasma from infected mice (day 13 PI) and from uninfected mice against proteins from *P*. *yoelii* 17XNL-infected and uninfected RBCs. Plasma from ABA-supplemented, infected mice contained higher levels of parasite-specific IgG1 and total IgG compared to unsupplemented, infected mice (Fig. 11A, Supplementary Fig. S4). Specifically, densitometry analysis revealed a 1.67-fold increase in levels of cross-reacting IgG1 and a 1.34-fold increase in levels of cross-reacting total IgG in plasma from ABA-supplemented, infected mice compared to unsupplemented, infected mice (Fig. 11B). Additionally, IgG1 from ABA-treated mice cross-reacted with at least one unique protein (~90 kDa) from infected RBCs that was not bound by IgG1 from unsupplemented, infected mice.

**Figure 12:**
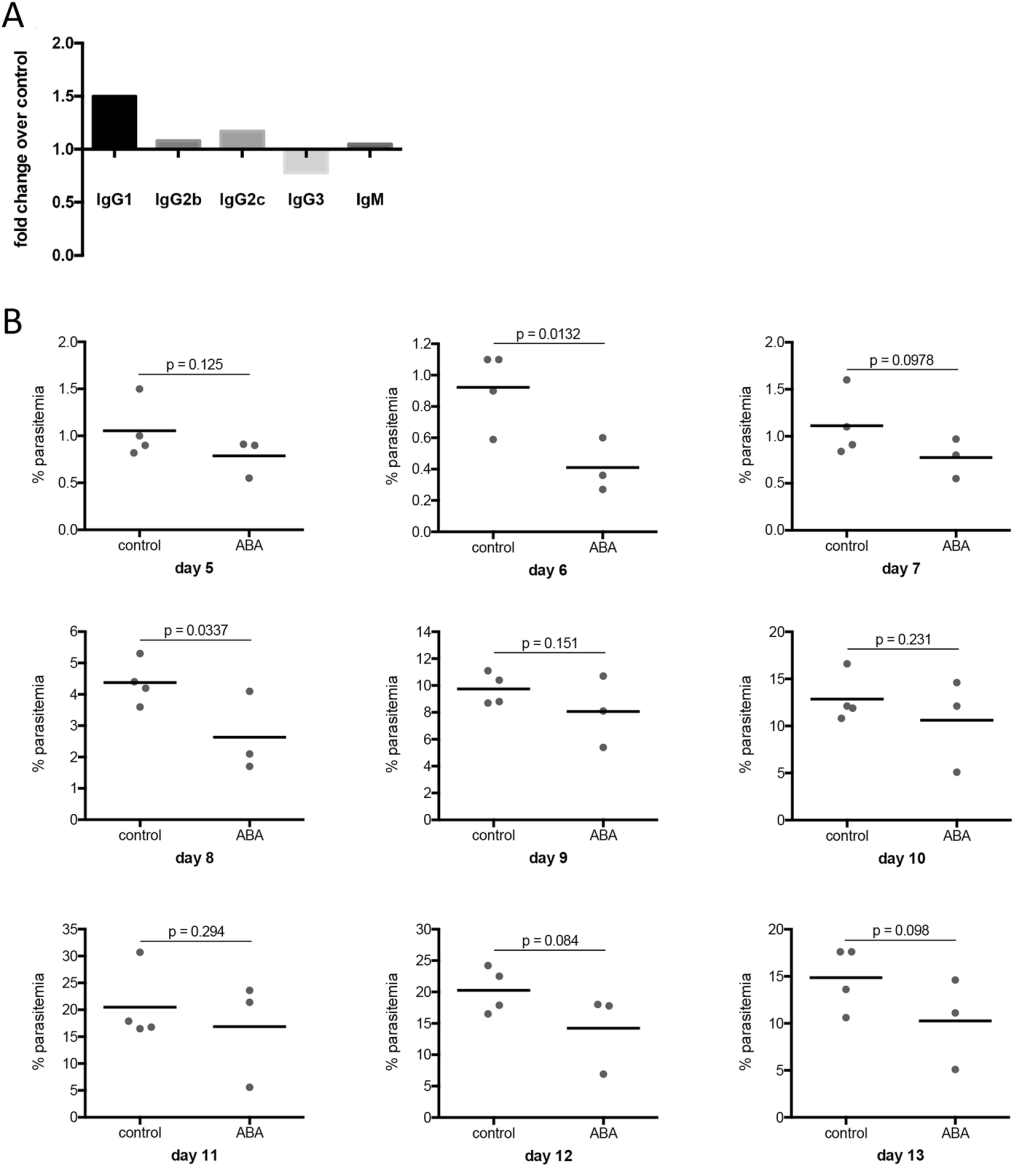
Plasma from ABA-supplemented mice exhibited limited protection against *P*. *yoelii* 17XNL infection. (**A**) Fold change in antibody isotype in pooled plasma from ABA-supplemented infected mice relative to unsupplemented, infected mice (day 13 PI). (**B**) Parasitemias of individual mice injected with plasma from unsupplemented, infected mice (control) or ABA supplemented, infected mice (ABA) days 5 through 13 PI. Data were analyzed by unpaired t-test. n = 3–4 mice per group.

To test whether enhance IgG levels were protective, mice were injected (tail vein) with 50 µl of plasma pooled from unsupplemented or ABA-supplemented, infected mice. Pooled plasma from ABA-supplemented mice used for passive immunization had 1.5-fold higher levels of IgG1 than that from control mice (Fig. 12A). At 24 hours after plasma injection, mice were infected with *P*. *yoelii* 17XNL. Mice that received plasma from ABA-supplemented, infected mice trended towards lower parasitemia and were significantly different than control-plasma injected mice on days 6 and 8 PI, suggesting that the plasma provided limited protection against infection (Fig. 12B), the duration of which was consistent with the half-life of IgG1 (6–8 days) in serum^44^.

### The effects of ABA on *P*. *yoelii* 17XNL parasitemia were ablated in caspase-1-deficient mice and this pattern was associated with changes in splenic and hepatic gene expression

**Figure 13:**
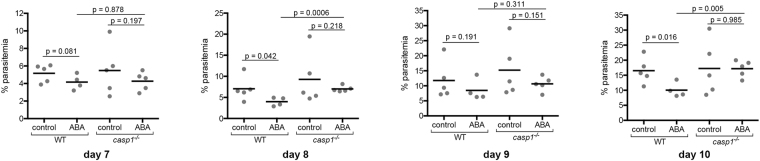
ABA supplementation did not reduce parasitemia in *caspase-1* knockout mice. Parasitemias of individual wild type (WT) and *casp1*^−/−^ mice with and without ABA supplementation from days 7 to 10 PI. 4–5 mice were used per treatment group. Data were analyzed by unpaired t-test.

Inflammasome activation in malaria results in the activation of caspase-1, which cleaves pro-IL-1β and IL-18 into their active forms^45^. To determine whether the effects of ABA on *P*. *yoelii* 17XNL infection were caspase-1-dependent, we examined parasitemia over time and hepatic and splenic gene expression on day 10 PI, when a majority of target mRNA levels were significantly different in ABA-supplemented and unsupplemented C57BL/6 mice (Figs 7A, 8A). In age-matched C57BL/6 mice, ABA supplementation significantly reduced parasitemia on days 8 and 10 PI (Fig. 13). However, while plasma levels of ABA in wild type and *casp1*^−/−^ mice were comparable (Fig. 4), ABA had no effect on parasitemia in *casp1*^−/−^ mice from days 7–10 PI (Fig. 13). Intriguingly, ABA supplemented wild type mice had significantly lower parasitemias than ABA-supplemented *casp1*^−/−^ mice on days 8 and 10 PI, while parasitemias of unsupplemented wild type and *casp1*^−/−^ mice did not significantly differ (Fig. 13). In the spleen, ABA-dependent decreases in *nos2*, *il6*, and *tbet* expression were ablated in *casp1*^−/−^ mice, while in *casp1*^−/−^ mice the ABA-dependent decrease in *ifnγ* was reversed and ABA treatment increased *pparγ* expression (Fig. 7B). In the liver, the ABA-dependent increase in *il6* was ablated in *casp1*^−/−^ mice and *il1β* was significantly reduced in ABA-treated *casp1*^−/−^ mice (Fig. 8B).

## Discussion

In our mouse malaria model, ABA supplementation has minimal to no effects in the absence of malaria and improves host health in the presence of parasite infection. In the absence of supplementation, infected mice do not generate detectable levels of plasma ABA (Fig. 4)^22^, so supplementation is necessary to study the effects of ABA on infection and pathology. Children with malaria, however, have plasma ABA levels consistent with those in supplemented outbred mice^22^ and elevated ABA levels were significantly associated with reduced risk of symptomatic malaria in the setting of *P*. *falciparum* infection. Given this clinical association, we sought to examine the mechanisms of ABA action in the context of malaria using a supplemented, genetically tractable mouse model.

We infer that no change or, at most, modest beneficial changes in CBC and plasma enzymes in supplemented mice are consistent with the hypothesis that ABA enhances anti-malarial immunity while mitigating inflammatory pathology. In particular, baseline glucose and insulin levels were not altered by ABA supplementation in either infected or uninfected mice, despite the fact that ABA can enhance insulin secretion *in vitro* and can reduce fasting glucose levels in a mouse model of diabetes^6,29^. These observations are consistent with the hypothesis that ABA supplementation does not exacerbate malaria-associated hypoglycemia and hyperinsulinemia.

Hepatic immunity clearly controls intrahepatic parasites, but the liver also contributes significantly to the control of erythrocytic asexual stages. These contributions include phagocytosis of iRBCs and secretion of the cytokines IL-6, TNFα, IL-18, and IL-1β^46^. IL-6 is typically more highly expressed in the liver than spleen^47^, suggesting that observed ABA-associated patterns in IL-6 expression warrant further study. IL-6 in the liver regulates the synthesis of acute phase proteins and contributes to the resolution of inflammation, the balance of which is dependent on classic and trans signaling by IL-6. In classic signaling, IL-6 binds a membrane bound IL-6 receptor (IL-6R), which interacts with the ubiquitous membrane protein gp130 to initiate JAK/STAT signaling and promote cell proliferation^48^. This signaling pathway is restricted to cells that express the bound IL-6R, including hepatocytes^37,38^. In trans signaling, IL-6 binds a soluble IL-6R, allowing it to bind to gp130 and initiate pro-inflammatory processes, most notably the synthesis of acute phase proteins (APP) in the liver. In the context of infection, inflammasome-associated IL-1β shifts IL-6-dependent synthesis of APP to those involved in pathogen defense, including hepcidin, C-reactive protein, serum amyloid P, and serum amyloid A^49^. Correlations between genetic mutations in *il1β* and *Plasmodium vivax* infection suggest that IL-1β is important for controlling parasitemia across *Plasmodium* species^50^. The ABA-dependent increase in liver *il6* was ablated in *casp1*^−/−^ mice and *il1β* was significantly reduced in ABA-treated *casp1*^−/−^ mice (Fig. 8B), suggesting that the balance of ABA-dependent host immunity and pathology in our model are caspase-1-dependent.

Increased levels of IgG1, IgG2, and IgG3 have been associated with malaria resistance and have been observed to be significantly higher in patients with uncomplicated and asymptomatic malaria compared to those with complicated malaria^51–54^. Similarly, T-bet-deficient mice had lower parasitemia, which correlated with increased levels of IgG1^55^. In our studies, plasma from ABA-supplemented mice provided limited protection against *P*. *yoelii* 17XNL infection. In a single tail vein injection, ABA levels in 50 µl plasma would be < 0.5 nM, providing confidence that the observed effects were due in part to elevated IgG. The half-life of IgG1 in serum is 6–8 days^44^, suggesting that a second injection of plasma post-infection could extend protection. IgG1 may enhance parasite clearance via antibody-dependent cell-mediated inhibition (ADCI), in which monocytes are activated by antibody-opsonized merozoites and release a soluble factor that blocks the division of intra-erythrocytic parasites^56^. Much lower levels of IgG1 are sufficient to trigger ADCI than is necessary for effective direct neutralization. Limited protection, however, also suggests that increased parasite-specific IgG is only one component of the protective response induced by ABA supplementation.

Caspase-1 deficiency abrogated the effect of ABA on parasitemia, suggesting that caspase-1 mediated immunity is critical to the reduction in parasitemia by ABA. Notably, the effects of ABA supplementation on *tbet* and *ifnγ* expression, which we hypothesize are driving protective IgG production, were eliminated or reversed in *casp1*^−/−^ mice (Fig. 7B). Caspase-1 also plays a role in IgG production during influenza infection. Specifically, caspase-1 knockout mice had reduced total IgG levels compared to wild type mice, but this was due mainly to reduced IgG3 levels and reductions in IgG1 levels were not observed^57^. The lack of effect of ABA supplementation on parasitemia in *casp1*^−/−^ mice suggests that inflammasome activation is the primary mechanism by which parasitemia is reduced. However, increased IgG may contribute to long-term immunity. Both the reduction in pathology and the enhanced IgG production in our model are consistent with the observations that higher plasma ABA concentrations were significantly associated with non-febrile falciparum malaria. Antibodies against *P*. *falciparum*-infected RBCs, as well as higher levels of IgG1, IgG2, and IgG3, have been associated with asymptomatic infection rather than symptomatic, febrile malaria^52,58^. Future work, however, is needed to determine whether ABA-enhanced antibody production provides protection against or enhances tolerance to infection.

## Methods

### Human plasma collection and ABA quantification

Plasma samples were selected from 122 *P*. *falciparum* infected children presenting for routine visits in the Nagongera and Kanungu surveillance cohorts of the East African International Center of Excellence in Malaria Research (ICEMR). The study protocol was reviewed and approved by the Uganda National Council of Science and Technology and the institutional review boards of the University of California–San Francisco, and Makerere University (DMID Protocol 10-0063). All experiments were performed in accordance with Good Clinical Practice (US Code of Federal Regulations,Ugandan Ethics Committee, and NIH/NIAID). Informed consent was obtained from the parent or guardian of all participating children. Samples were selected based on age stratification (2–4 and 7–9 year-olds), and log parasite density (range: 32 to >100,000 parasites/µL). Parasitemia at routine visits was determined and quantified by microscopy of thick blood smears, as previously described^23^. Symptomatic subjects were defined as those with fever at the time of the visit or with a reported history of fever. ABA was extracted from plasma as previously described^22^ and quantified using the Phytodetek Abscisic Acid ELISA kit (Agdia, Elkhart, IN) according to the manufacturer’s instructions. ABA levels were analyzed by unpaired t-test.

ABA was quantified in mouse plasma by LC-MS/MS using an AB Sciex API2000 tandem mass spectrometer coupled with PE 200 series micro LC pumps (calibration range 25–10,000 ng/mL) or API5000 tandem mass spectrometer coupled with Shimadzu Prominence 20AD^XR^ LC pumps (calibration range 0.5–50 ng/mL). ABA was extracted from EDTA mouse plasma samples by solid phase extraction with Oasis® HLB micro-elution plate. Extracted samples were injected onto a Zorbax C_8_ LC column and eluted with 10 mM NH_4_FA-acetonitrile containing 0.1% formic acid. Electrospray ionization in negative mode and multiple reaction monitoring were used, and ion pairs m/z 263- >153 was selected for quantification. ABA levels were analyzed by unpaired t-test.

### ABA supplementation and infection

Mice received control or 2.56 mM ABA-supplemented water three days prior to intraperitoneal (IP) injection with 1 × 10^7^ *P*. *yoelii* 17XNL-iRBCs as described previously^22^. Parasitemias were determined from thin blood films stained with Giemsa and were defined as the percentage of iRBCs divided by total RBCs. Mice were sacrificed on days 9, 10, 11 and 13 PI. Parasitemia was analyzed by unpaired t-test. Hepatic and splenic gene expression data were obtained from CD-1 mice with previously quantified parasitemias^22^. *Casp1*^−/−^ mice were obtained from Jackson Labs. Age-matched (6–8 week old) wild type C57BL/6 mice (Jackson Labs) were used as controls. All experiments were performed in accordance with the recommendations in the Guide for Care and Use of Laboratory Animals of the National Institute of Health and were approved by the Institutional Animal Care and Use Committees at the University of California at Davis under protocol 18948.

### Blood analysis

Blood was collected from individual mice via heart puncture with heparinized needles on day 11 PI. Whole blood was collected in heparinized tubes and CBC performed using a HemaVet 950 FS (Drew Scientific). Blood was centrifuged at 5,900 × g for 8 min for plasma collection. Aliquots of plasma were stored at −20 °C and insulin levels determined using the Ultra Sensitive Mouse Insulin ELISA Kit (Crystal Chem #90080) according to the manufacturer’s instructions. Blood cell counts and analytes were analyzed by unpaired t-test.

### qRT-PCR

Harvested liver and spleen were flash frozen and stored at −80 °C. RNA was extracted in TriZOL and complementary DNA (cDNA) synthesized according to manufacturer’s instructions (QuantiTect Reverse Transcription Kit, Qiagen). Quantitative real time polymerase chain reaction (qRT-PCR) was performed using validated TaqMan gene expression assays as previously described for mouse *β-actin*, *nos2*, *tnfα*, *ifnγ*, *pparγ*, *il6*, *il18*, *il1β*, and *tbet* (Applied Biosystems)^59^. Samples were analyzed in triplicate using 250 ng cDNA per reaction. Data were normalized to *β-actin* and analyzed by unpaired t-test. Outliers were tested by ROUT with Q = 2%.

### Antibodies and plasma injection

Antibody levels in mouse plasma were determined using the ProcartaPlex Mouse Antibody Isotyping Panel (Affymetrix eBioscience EPX070-20815-901) according to the manufacturer’s instructions. Day 13 PI plasma from individual mice was pooled. C57BL/6 mice received 50 µL of plasma from control or ABA-supplemented infected mice by tail-vein injection. At 24 hours after plasma injection, mice were infected IP with 1 × 10^7^ *P*. *yoelii* 17XNL-infected RBCs.

### Western blot

Blood was collected from uninfected and *P*. *yoelii* 17XNL*-*infected mice and centrifuged at 2,000 × g for 10 min at room temperature to isolate RBCs. Equivalent volumes of collected RBCs were washed twice with RPMI medium before being suspended in lysis buffer. RBC proteins were separated by gel by electrophoresis, transferred to nitrocellulose, and incubated overnight at 4 °C in 5% dry milk in Tris-buffered saline, 0.1% Tween 20 (TBST). Gels were stained with Coomassie to quantify total protein. Membranes were incubated for 2 hours at room temperature with 1:100 dilutions of plasma from uninfected mice, from infected mice at day 13 PI, or from ABA-treated infected mice at day 13 PI. Washed membranes were then incubated with 1:1000 goat anti-mouse IgG1 (Abcam #ab97240) or 1:1000 rabbit anti-mouse IgG (Sigma #A9044) HRP-conjugated secondary antibodies for 1 hour at room temperature.

### Data availability

All data generated or analyzed during this study are included in this published article and its Supplementary Information files.

## Electronic supplementary material

Supplemental Figures
