# Supplementary material for: Elevated plasma abscisic acid is associated with asymptomatic falciparum malaria and with IgG-/caspase-1-dependent immunity in Plasmodium yoelii-infected mice
Source: Sci Rep. 2018 Jun 11;8:8896. doi: 10.1038/s41598-018-27073-1 (PMC5995817; doi:10.1038/s41598-018-27073-1)
Supplement: Supplementary file 1 — Supplemental Figures [file 41598_2018_27073_MOESM1_ESM.pdf]

Elevated plasma abscisic aid is associated with asymptomatic falciparum malaria and with IgG-/caspase-1-dependent immunity in *Plasmodium yoelii*-infected mice

Elizabeth K.K. Glennon[1,a], Dewi Megawati[1,b], Brandi K. Torrevillas[1,b], Isaac Ssewanyana[2,3], Liusheng Huang[4], Fran Aweeka[4], Bryan Greenhouse[5], L. Garry Adams[6], Shirley Luckhart[1,b,*]

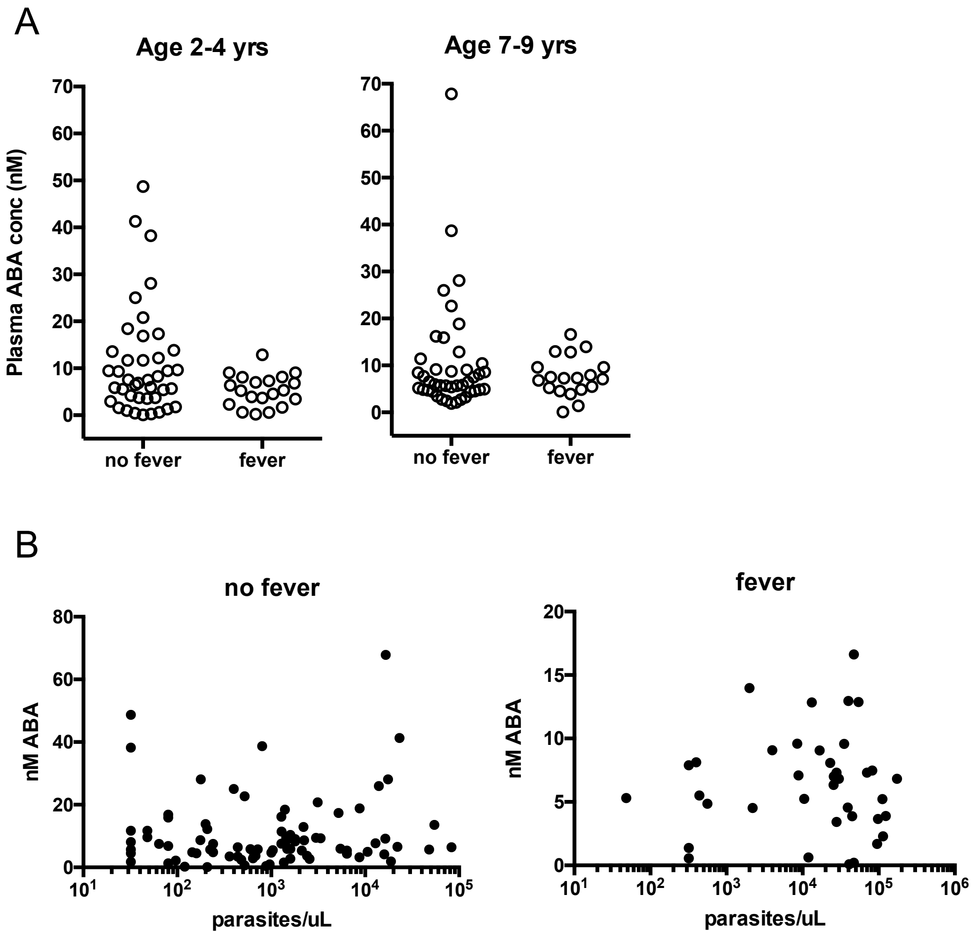

Supplementary Figure S1. Children with malaria without fever had higher associated plasma ABA levels. (A) Plasma ABA levels in children infected with *P. falciparum* separated by age group. (B) Plasma ABA levels plotted against parasitemia at the time of blood collection for individuals presenting with and without fever. Each dot represents one individual.

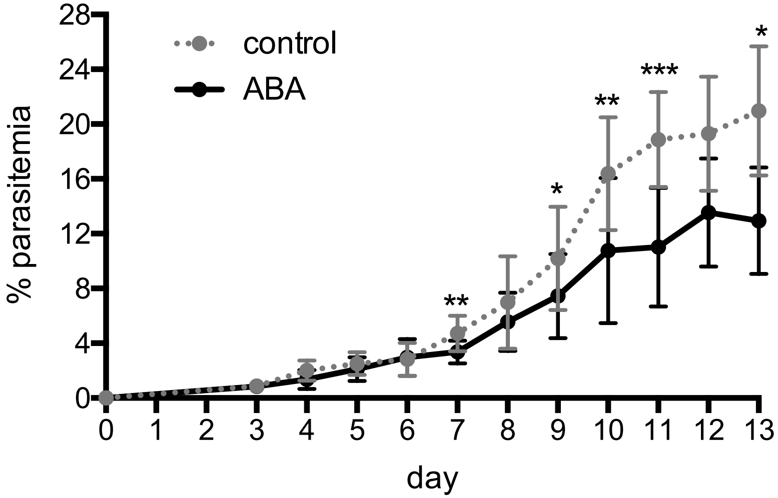

Supplementary Figure S2. ABA supplementation significantly reduced parasitemia in C57BL/6 mice infected with *P. yoelii* 17XNL. Parasitemias of mice with and without oral ABA supplementation from day 4 to 13 post-infection. Four replicates were conducted with 3-5 mice per treatment in each replicate. Mice were sacrificed for analyses on days 9, 11 and 13, reducing animal numbers after these days. Data were analyzed by unpaired t-test. * $p < 0.05$ ** $p < 0.01$ *** $p < 0.0001$

	Control		ABA			*P. yoelii*		*P. yoelii* + ABA		
	Mean	SE	Mean	SE	p value	Mean	SE	Mean	SE	p value
albumin (g/dL)	3.14	0.13	3.23	0.29	0.78	2.55	0.18	2.25	0.37	0.46
blood urea nitrogen (mg/dL)	28	2.5	28.76	2.25	0.83	20.94	0.95	26.00	2.43	0.036
creatine (mg/dL)	0.17	0.01	0.21	0.03	0.13	0.16	0.01	0.18	0.02	0.45
phosphorus (mg/dL)	9.69	0.42	10.07	1.00	0.72	8.61	0.42	8.43	0.31	0.75
total bilirubin (mg/dL)	0.08	0.03	0.06	0.01	0.5	0.25	0.03	0.26	0.04	0.82
total protein (g/dL)	4.24	0.2	4.67	0.26	0.21	4.41	0.2	4.12	0.41	0.51

Supplementary Table S1. Blood chemistry panel. ABA supplementation in uninfected and *P. yoelii* 17XNL-infected mice had no effect on plasma indicators of organ health. n = 4-5 mice per treatment group. Data were analyzed by unpaired t-test.

	Control		ABA			*P. yoelii*		*P. yoelii* + ABA		
	Mean	SE	Mean	SE	p value	Mean	SE	Mean	SE	p value
WBC (K/uL)	5.66	0.76	5.73	0.63	0.94	31.27	0.29	31.90	3.63	0.89
% neutrophils	15.35	2.01	17.53	3.97	0.64	18.4	1.9	22.8	3.56	0.28
% lymphocytes	69.89	3.2	66.56	3.94	0.53	75.64	2.02	69.22	4.37	0.19
% monocytes	10.28	0.89	11.71	0.94	0.3	4.56	0.39	5.31	0.73	0.37
RBCs (M/ul)	8.73	0.3	8.94	0.7	0.8	3.97	0.26	3.83	0.31	0.72
hemoglobin (g/dL)	11.7	0.52	12.56	0.86	0.42	7.46	0.26	7.28	0.37	0.69
% hematocrit	36.88	1.56	38.48	2.76	0.63	22.02	0.97	21.00	1.36	0.55
MCV (fL)	42.22	1.18	43.14	0.65	0.52	55.76	1.55	55.20	1.57	0.81
MCH (g/dL)	13.4	0.19	14.1	0.32	0.08	18.92	0.59	19.18	0.67	0.78
MCHC (g/dL)	31.76	0.87	32.72	0.75	0.43	33.94	0.49	34.73	0.54	0.32
% RDW	16.8	0.42	17.02	0.24	0.66	37.14	0.99	34.95	2.07	0.34
platelets (K/ul)	487.4	89.42	573.0	134.5	0.61	278.0	14.8	234.3	23.48	0.14
MPV (fL)	5.52	0.12	5.74	0.15	0.29	6.46	0.07	6.23	0.11	0.11

Supplementary Table S2. Complete blood count. ABA supplementation in uninfected and *P. yoelii* 17XNL-infected mice had no effect on whole blood composition. n = 4-5 mice per treatment. Data were analyzed by unpaired t-test. WBC = white blood cells, RBCs = red blood cells, MCV = mean corpuscular volume, MCH = mean corpuscular hemoglobin, MCHC = mean corpuscular hemoglobin concentration, RDW = red blood cell distribution width, MPV = mean platelet volume.

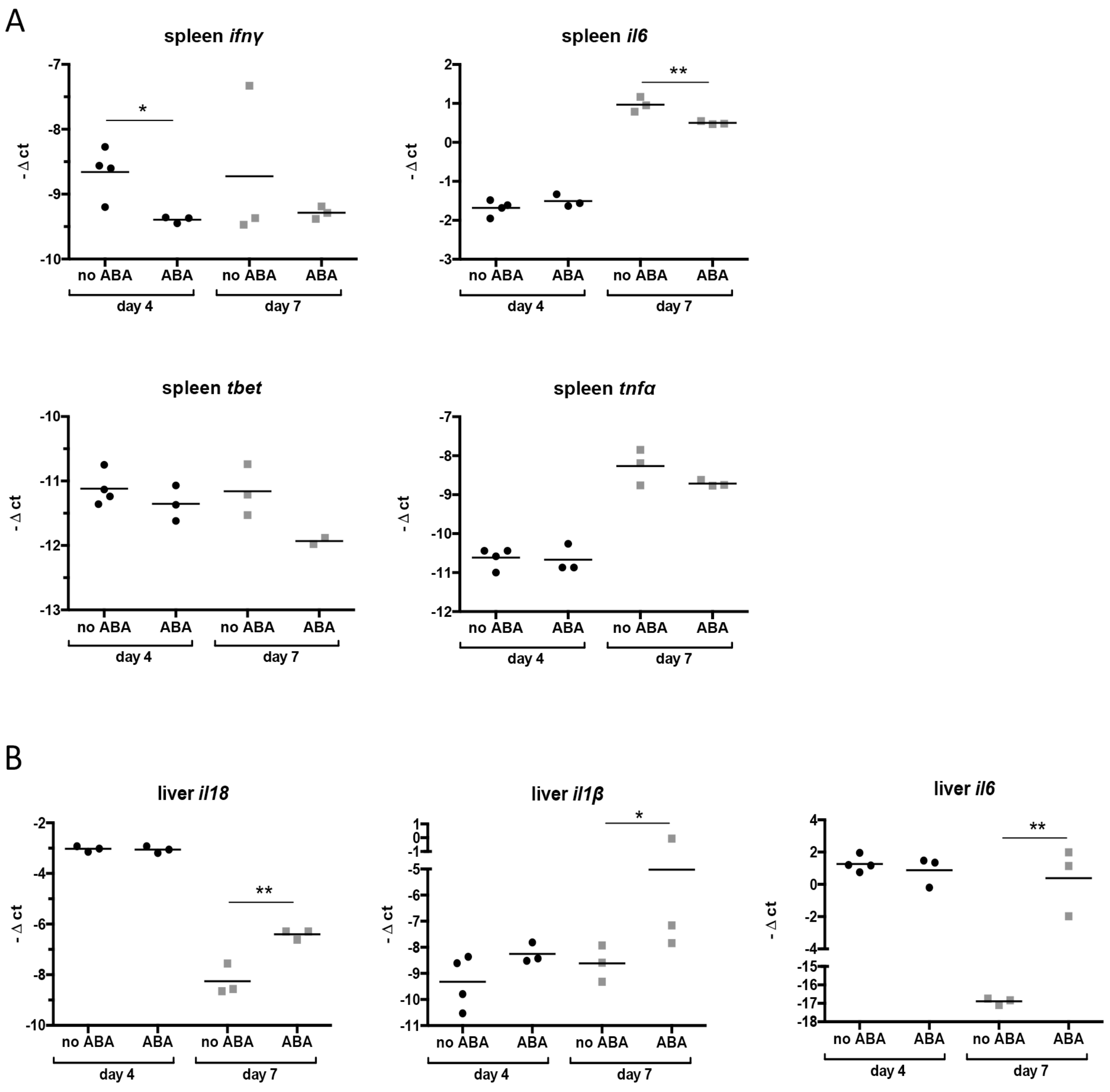

Supplementary Figure S3. Hepatic and splenic gene expression patterns in CD-1 mice following ABA supplementation were similar to those in C57BL/6 mice (Figs 7 and 8). Relative mRNA levels of gene targets in the spleen (A) and liver (B) of unsupplemented and ABA-supplemented CD-1 mice on days 4 and 7 PI with *P. yoelii* 17XNL. Data are shown as –Δct, normalized to *β-actin* and were analyzed by unpaired t-test. Each dot represents one mouse. * $p < 0.05$, ** $p < 0.01$.

A

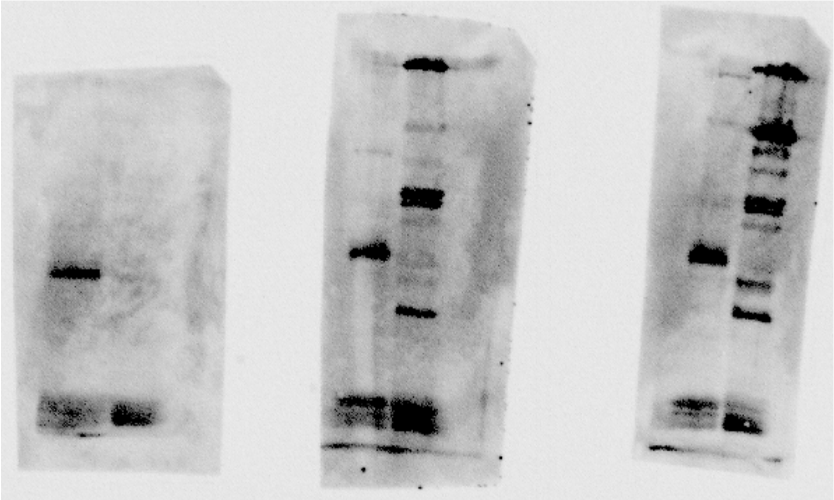

B

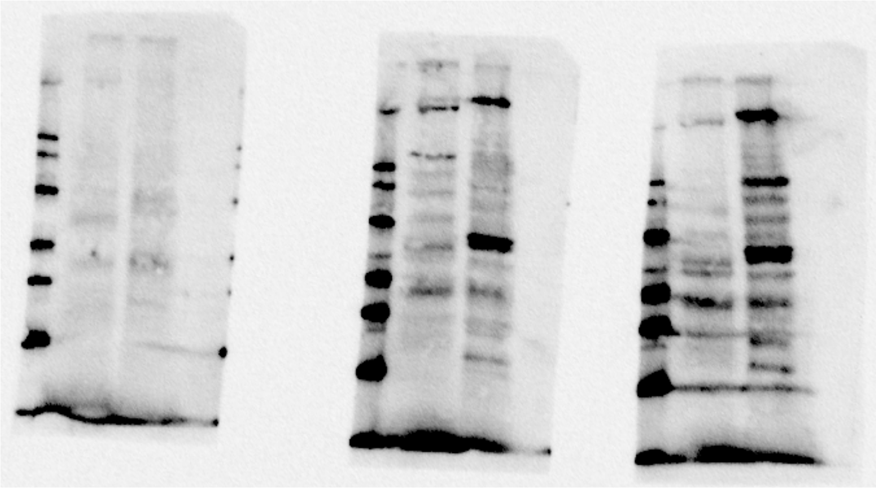

Supplementary Figure S4. Original western blot images of protein from uninfected and *P. yoelii* 17XNL-infected RBCs probed with plasma from uninfected mice, *P. yoelii*-infected mice or ABA-supplemented *P. yoelii*-infected mice followed by secondary antibodies to (A) IgG1 and (B) total IgG.
